# The Reciprocal Causation of the ASK1-JNK1/2 Pathway and Endoplasmic Reticulum Stress in Diabetes-Induced Cognitive Decline

**DOI:** 10.3389/fcell.2020.00602

**Published:** 2020-07-17

**Authors:** Yanqing Wu, Yuan Yuan, Chengbiao Wu, Ting Jiang, Beini Wang, Jun Xiong, Peipei Zheng, Yiyang Li, Jingyu Xu, Ke Xu, Yaqian Liu, Xiaokun Li, Jian Xiao

**Affiliations:** ^1^The Institute of Life Sciences, Engineering Laboratory of Zhejiang Province for Pharmaceutical Development of Growth Factors, Biomedical Collaborative Innovation Center of Wenzhou, Wenzhou University, Wenzhou, China; ^2^Research Units of Clinical Translation of Cell Growth Factors and Diseases Research of Chinese Academy of Medical Science, School of Pharmaceutical Science, Wenzhou Medical University, Wenzhou, China; ^3^Clinical Research Center, Affiate Xiangshang Hospital, Wenzhou Medical University, Wenzhou, China

**Keywords:** diabetes-induced cognitive decline (DICD), hippocampus, neuronal apoptosis, apoptosis signal-regulating kinase 1 (ASK1), endoplasmic reticulum (ER) stress

## Abstract

Diabetes significantly induces cognitive dysfunction. Neuronal apoptosis is the main cause of diabetes-induced cognitive decline (DICD). Apoptosis signal-regulating kinase 1 (ASK1) and endoplasmic reticulum (ER) stress are remarkably activated by diabetes. The role and relationship of ASK1-JNK1/2 signaling and ER stress in DICD have not yet been elucidated. In this study, we used db/db mice as the DICD animal model and confirmed that db/db mice displayed cognitive decline with inferior learning and memory function. Diabetes significantly induced morphological and structural changes, excessive neuronal apoptosis, Aβ_1__–__42_ large deposition, and synaptic dysfunction in the hippocampus. Mechanistic studies found that diabetes significantly triggered ASK1-JNK1/2 signaling activation and increased ER stress in the hippocampus. Moreover, diabetes enhanced the formation of the IRE1α–TRAF2–ASK1 complex, which promotes the crosstalk of ER stress and the ASK1-JNK1/2 pathway during DICD. Furthermore, 4-PBA treatment blocked high glucose (HG)-induced ASK1-JNK1/2 signaling activation, and excessive apoptosis *in vitro*. Inhibiting ASK1 via siRNA remarkably ameliorated the HG-induced increase in p-IRE1α and associated apoptosis in SH-SY5Y cells, suggesting that ASK1 is essential for the assembly and function of the proapoptotic kinase activity of the IRE1α signalosome. In summary, ER stress and ASK1-JNK1/2 signaling play causal roles in DICD development, which has crosstalk through the formation of the IRE1α–TRAF2–ASK1 complex.

## Introduction

Diabetes is a serious, chronic metabolic disorder that adversely affects multiple organs due to its long-term complications, and the brain is one of the major targets, which results in diabetic encephalopathy (DE) (Mijnhout et al., 2006). Diabetes-induced cognitive decline (DICD) is one of the most common types of DE. The duration of diabetes and level of hyperglycemia are positively correlated with the level of cognitive dysfunction (Li et al., 2003; de la Monte and Wands, 2008). The hippocampus, which is highly susceptible to hyperglycemia, is essential for short-term memory, learning, executive ability, and attention of brain. The cornu ammon1 (CA1) region in the hippocampus is most closely related to cognitive function, and thus considered as a specific target for the changes related to cognitive function in DICD studies (Di Mario et al., 1995; Sun et al., 2014). Although DICD has attracted extensive attention, the molecular mechanisms underlying it are not well understood.

Excessive apoptosis is one of the main factors that causes diabetes-mediated complications. Increasing evidence has suggested that hyperglycemia-induced neuronal apoptosis in the hippocampus plays a crucial role in DICD development (Kroemer, 1997; Yan, 2014). It has been reported that diabetes triggers elevated endoplasmic reticulum (ER) stress and apoptosis signal-regulating kinase 1 (ASK1) signaling activation, which induces the apoptotic cascade (Wang et al., 2015a; Wang et al., 2015b). The ER is responsible for correct folding of secretory and transmembrane proteins. The accumulation of unfolded or misfolded proteins triggers the adaptive unfolded protein response (UPR) by activating these sensors: including inositol-requiring enzyme 1α (IRE1α), protein kinase RNA-like ER kinase (PERK), and activated transcription factor 6a (Ron and Walter, 2007). Persistent UPR results in severe ER stress, and consequently induces cell damage or even apoptosis (Shore et al., 2011). Increasing evidence has shown that ER stress-triggered apoptosis participates in the occurrence and progression of neurodegenerative disease (Wei et al., 2014; Wang et al., 2016; Lyra et al., 2019).

ASK1, a proapoptotic kinase, is an oxidative stress-responsive kinase, whose activation leads to the phosphorylation of JNK1/2 and transmits the proapoptotic cascade signaling to the nucleus. ASK1 is a crucial facilitator and therapeutic target for preventing brain injury associated with obesity (Toyama et al., 2015). Excessive oxidative stress in the hippocampus has been reported as a critical contributing factor for diabetes-induced cognitive dysfunction (Davari et al., 2013; Adedara et al., 2019; Gocmez et al., 2019). However, it is unknown whether ASK1-JNK1/2 signaling is involved in diabetes-triggered hippocampal neuronal apoptosis, and its mutual regulatory relationship with ER stress during DICD is also not well understood.

IRE1α has both protein kinase and endoribonuclease (RNase) activities (Nishitoh et al., 2002; Han et al., 2009; Wang et al., 2015a). Once apoptosis is initiated, IRE1α interacts with TNF receptor-associated factor 2 (TRAF2) and ASK1, forming a proapoptotic signalosome, the IRE1α–TRAF2–ASK1 complex (Urano et al., 2000; Nishitoh et al., 2002). ER stress activates ASK1-JNK1/2 signaling and subsequently triggers the mitochondrial apoptotic pathway through the formation of this complex (Hetz et al., 2006; Chen et al., 2014). Moreover, ASK1 is a key component in the UPR signalosome that leads to ER stress (Urano et al., 2000; Nishitoh et al., 2002). Thus, we hypothesize that the ASK1-JNK1/2 pathway is involved in diabetes-induced neuronal apoptosis in the hippocampus, which has a crosstalk with ER stress through the formation of the IRE1α–TRAF2–ASK1 complex during DICD.

In the current study, we used db/db mice as the DICD animal model and investigated the role of ASK1-JNK1/2 signaling and its relationship with ER stress of neuronal apoptosis in the hippocampus during DICD. This study aimed to clarify the molecular mechanisms underlying DICD and offer a novel theoretical basis for DICD treatment.

## Materials and Methods

### Animal and Experimental Design

Twenty-week-old male db/db (C57BLKS/J-leprdb/leprdb) mice and their non-diabetic db/m littermates were purchased from the Model Animal Research Center of Nanjing University (Nanjing, China). The animals were maintained under a 14-h light/10-h dark condition. After arrival, the animals were acclimatized to animal house for 2 weeks. Then, the mice were performed the Morris water maze test. After the Morris water maze test, they were anesthetized with 10% chloral hydrate (3.5 ml/kg). For histomorphological analysis, the animals were perfused with 4% paraformaldehyde (PFA) in 0.1 M phosphate-buffered saline (PBS) following the saline solution perfusion, and then the brains were rapidly detached and post-fixed by immersion in 4% PFA. For molecular biological analysis, the hippocampus was separated from the brain after perfusion with 0.9% saline solution and rapidly stored at −80°C.

### Morris Water Maze Test

The test was performed in a circular pool with a diameter of 120 cm and a height of 40 cm (Jiliang, Shanghai, China). It was filled with opaque water colored with milk powder and maintained at a temperature of 26 ± 1°C. Using a hidden circular platform, the training was carried out with six blocks that consisted of three 60-s trials separated by 20-min inter-block intervals as previously described (Nicholas et al., 2006; Xu et al., 2015). During the training, the platform remained in the same location relative to the distal cues in the room. For each trial, mice were placed in the water at different start locations (E, S, W, and N) that were equally spaced from each other and were offset from the goal location by 45°. One hour following the sixth block, the hidden platform was removed, and the mice were scored during a 60-s probe trial. They were scored for latency to reach the goal and for memory recall, which was determined by crossing over the previous platform location. Another probe trial was performed 24 h after training to assess memory consolidation and memory retrieval.

### Hematoxylin and Eosin (H&E) Staining and Nissl Staining

The brains were collected and fixed with 4% PFA in PBS. Then, the brains were dehydrated in alcohol and embedded with paraffin. After that, 5-μm sections were dewaxed and hydrated, then stained with hematoxylin and eosin solutions (Solarbio Science and Technology, Beijing, China), and observed under light microscope. For Nissl staining, tissue sections were stained with cresol violet and Nissl differentiation solutions according to the instructions (Beyotime, Shanghai, China), and observed under Nikon ECLPSE 80i (Nikon, Tokyo, Japan).

### Immunohistochemical Staining

After dewaxing and hydration, the brain sections were incubated in 3% H_2_O_2_ for 15 min, and then in blocking solution for 45 min. Subsequently, the sections were incubated with the following primary antibodies at 4°C overnight: Aβ_1__–__42_ (1:400, Abcam, Cambridge, United Kingdom). After washing three times in PBS, the sections were incubated with horseradish peroxidase-conjugated secondary antibodies for 4 h at 37°C. Then, the sections were reacted with 3,3-diaminobenzidine (DAB) and imaged under Nikon ECLPSE 80i (Nikon, Tokyo, Japan).

### Immunofluorescent Staining

After dewaxing and hydration, the brain sections were incubated in 3% H_2_O_2_ for 15 min, and then incubated with 5% bovine serum albumin (BSA) in a 37°C oven for 30 min. Then, the sections were incubated with the following primary antibody at 4°C overnight: p-Tau (1:200, abcam). After triple washing in PBS at room temperature, the sections were once again incubated with Alexa Fluor 647 (1:1000, Abcam) as secondary antibody for 4 h. Fluorescence images were captured using a Nikon ECLPSE 80i (Nikon, Tokyo, Japan).

### Immunoprecipitation and Western Blotting Analysis

The hippocampus was separated from the brain soon after the mice were sacrificed, and it was stored at −80°C for subsequent analysis. For protein extraction, the hippocampus was homogenized in lysis buffer containing protease inhibitor cocktail (10 μl/ml, GE Healthcare Biosciences, PA, United States). Then, the complex was centrifuged at 12,000 rpm, and the supernatant was obtained for the protein assay. The extracted protein was quantified with BCA reagents (Beyotime). For IP, 300 mg of protein from hippocampus was incubated with 1 mg of rabbit anti-ASK1 antibody at 4°C overnight. After that, the protein was further incubated with protein A magnetic bead slurry. Then, the protein was separated on a 10% or 12% gel, and transferred onto a PVDF membrane (Sigma-Aldrich, St Louis, MO). The membrane was blocked with 5% milk in TBS for 0.5 h and incubated with primary antibodies in TBS overnight at 4°C. After washing three times with TBST (TBS with 0.05% tween 20), the membrane was treated with horseradish peroxidase-conjugated secondary antibodies (1:3000) for 4 h at room temperature. Signals were visualized by ChemiDocXRS + Imaging System (BioRad). All experiments were repeated in triplicate using independently prepared tissue. The densitometric values of bands on Western blotting were obtained by Image J software and subjected to statistical analysis.

### TUNEL Staining

TUNEL staining was performed using the ApopTag Fluorescein Direct *In Situ* Apoptosis Detection Kit (Roche, Basel, Switzerland). According to the standard protocol, after dewaxing and hydration, the brain sections or cells were incubated with 20 μg/ml proteinase K working solution for 15 min at 37°C. The slides were then rinsed three times with PBS, which was followed by incubation with the TUNEL reaction mixture for 1 h at 37°C. After rinsing three times with PBS, the sections or cells were treated with 4′,6-diamidino-2-pheny-lindole (DAPI, Beyotime) for 5 min at room temperature and mounted with aqueous mounting medium. The results were imaged under a Nikon ECLIPSE 80i microscope.

### SH-SY5Y Cells Culture and Treatment

SH-SY5Y cells were purchased from the Cell Storage Center of Wuhan University (Wuhan, China). SH-SY5Y cells were cultured in Dulbecco’s Modified Eagle Medium (DMEM, Gibco, United States) supplemented with 10% fetal bovine serum (FBS, Gibco, United States) and antibiotics (100 units/ml penicillin and 100 μg/ml streptomycin). They were incubated in a humidified atmosphere containing 5% CO_2_ at 37°C. Either glucose or mannitol was added as the high glucose (HG) group or the osmotic control, respectively. CCK8 assay (Beyotime, Shanghai, China) was used to detect the optimum concentration of HG. After seeding for 24 h, the cells were cultured in HG (100 mM) media with or without 2 mmol/L ER stress inhibitor 4-phenylbutyric acid (4-PBA) to inhibit ER stress for 24 h. Additionally, ASK1 small interfering RNA (ASK1-siRNA; sc-29749, Santa Cruz Biotechnology, CA, United States) and control siRNA (sc-37007) were used to inhibit ASK1 expression in cells for 24 h. Lipofectamine RNAiMAX (Santa Cruz Biotechnology) was used according to the manufacturer’s protocol for transfection of siRNA into the cells.

### Statistical Analyses

Data were presented as means ± SEM. Experiments were repeated at least three times, and hippocampus from each replicate was from different mice. Statistical differences were determined by one-way analysis of variance (ANOVA) using GraphPad Prism 5. In one-way ANOVA analysis, Tukey test was used to estimate the significance of the results (*p* < 0.05). Statistical significance was accepted when *p* < 0.05.

## Results

### Diabetes Significantly Induces Cognitive Decline of Mice

**FIGURE 1:**
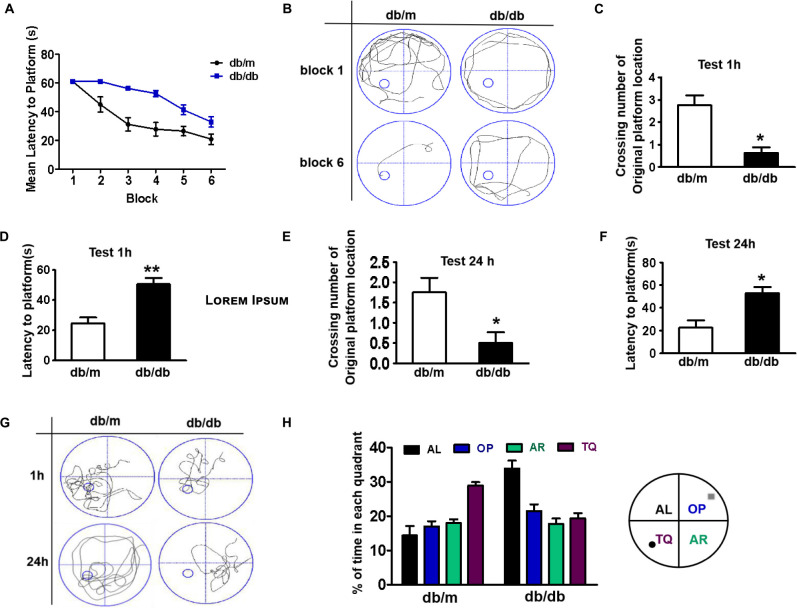
Diabetes significantly induces cognitive decline of mice. **(A)** The learning curve of the training period of mice during six blocks in the Morris water maze test. **(B)** Representative swimming track of mice at block 1 and block 6 during the training period. **(C)** Number of crossings over the original platform location of mice in the probe trial (1 h after training). **(D)** Latency to find the platform of mice in the probe trial (1 h after training). **(E)** Number of crossings over the original platform location of mice in the probe trial (24 h after training). **(F)** Latency to find the platform of mice in the probe trial (24 h after training). **(G)** Representative swimming track of mice in the probe trial (1 and 24 h after training). **(H)** Percentage of residence time in each quadrant. The quadrant with the platform was designated as TQ and the quadrant from which the mice started their swimming was designated as OP for “opposite”. The quadrant on the left side of OP was designated as AL for “adjacent left” and the quadrant on the right side of OP was designated as AR for “adjacent right”. *p < 0.05, **p < 0.01 vs db/m, *n* = 10.

The mice were trained to learn how to locate the platform throughout six blocks, and then performed the test after 1 or 24 h of training. There was a significant difference in the latency of db/db mice to reach the platform during the six training blocks when compared with that of db/m mice. The db/db mice took longer and had poor orientation to reach the platform during training, suggesting that db/db mice had inferior learning ability (Figures 1A,B). After 1 h of training, we had removed the platform and further tested the difference in the spatial memory ability of the mice in a probe trial. It was observed that db/db mice had fewer number of crossings over the platform position and took longer to reach the platform than db/m mice (Figures 1C,D, *p* < 0.05). After 24 h, memory retention of the platform location was still worse for db/db mice, as indicated with fewer crossing numbers over the platform and taking longer to reach the platform (Figures 1E,F, *p* < 0.05). Additionally, the swimming track and the retention time in the target quadrant of the platform of mice during the trial had further indicated that db/db mice have worse memory function than db/m mice (Figures 1G,H). Taken together, these results suggest that diabetes significantly induces inferior learning and memory function of mice.

### Diabetes Significantly Leads to Morphological Changes, Aβ_1__–__42_ Deposition, Hyperphosphorylation of Tau, and Synaptic Dysfunction in the Hippocampus During DICD

**FIGURE 2:**
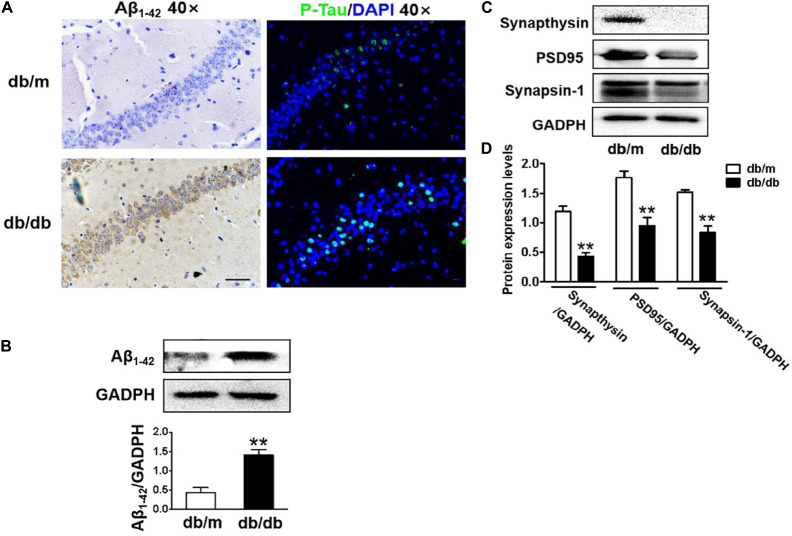
Diabetes significantly leads to morphological changes, Aβ_1__–__42_ deposition, hyperphosphorylation of Tau and synaptic dysfunction in the hippocampus during DICD. **(A)** The representative images of H&E staining, Nissl staining, immunohistochemical staining of Aβ_1__–__42_, and immunofluorescence staining of p-Tau in the CA1 region of the hippocampus of db/m mice and db/db mice (scale bar = 15 μm). **(B)** Westere blotting and quantitative analysis of Aβ_1__–__42_ in the hippocampus of db/m mice and db/db mice. **(C,D)** Western blotting and quantitative analysis of synaptic function-related protein expression (PSD95, synaptophysin, and synapsin-1) in the hippocampus of db/m mice and db/db mice. *p < 0.05, **p < 0.01 vs db/m mice, *n* = 3.

In this study, the neuronal cells in the CA1 region of the hippocampus of db/db mice exhibited an extensive loss, karyopyknosis, unclear cell membranes, and sparse arrangement (Figure 2A). Aβ_1__–__42_ deposition is a characteristic of Alzheimer’s disease. Thus, we detected the expression of Aβ_1__–__42_ in the hippocampus and found that Aβ_1__–__42_ was largely deposited in the CA1 region of the hippocampus in db/db mice when compared with that in db/m mice (Figures 2A,B). Moreover, phosphorylated Tau levels in the CA1 region of the hippocampus in db/db mice were also remarkably higher than those in db/m mice (Figure 2A). Additionally, we further explored the role of hyperglycemia in synaptic function and observed that the expression of synaptic function-related proteins (PSD95, synaptophysin, and synapsin-1) in the hippocampus was remarkably suppressed under hyperglycemia (Figures 2C,D).

### Diabetes Results in Excessive Neuronal Apoptosis of Hippocampus

**FIGURE 3:**
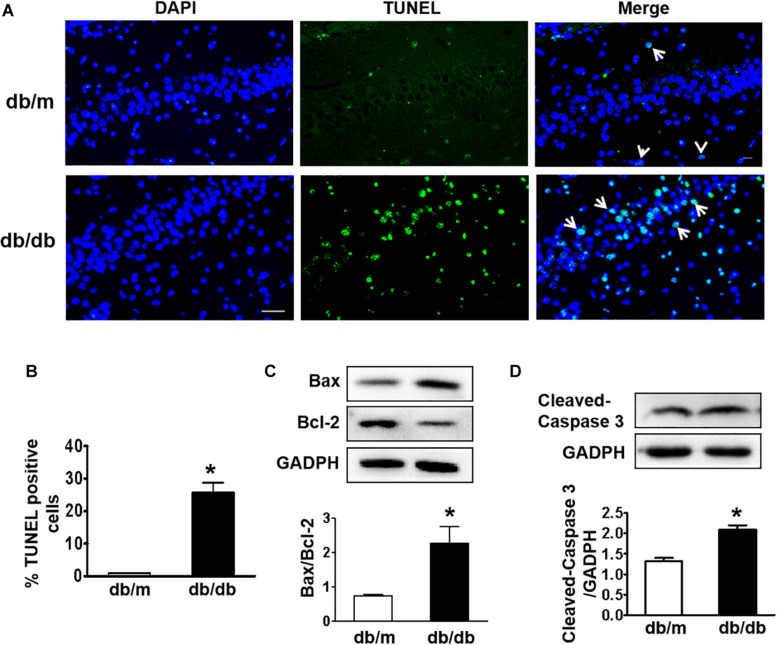
Diabetes triggers neuronal apoptosis in the hippocampus. **(A)** Representative images of the TUNEL assay showing apoptotic cells (green signal) in the CA1 region of the hippocampus. Cell nuclei were stained with DAPI (blue) (scale bar = 15 μm). **(B)** The quantificative analysis of TUNEL-positive cells in the CA1 region of the hippocampus. **(C,D)** Western blotting and quantitative analysis of Bax, Bcl-2, and cleaved caspase-3 expression in the hippocampus of db/m and db/db mice. *p < 0.05 vs db/m mice, *n* = 3.

Next, we used the TUNEL assay to test whether excessive cell apoptosis is involved in DICD development. The number of TUNEL-positive cells in the CA1 region of hippocampus from db/db mice was much greater than that in db/m mice (Figures 3A,B). Diabetes significantly decreased Bcl-2 expression and enhanced Bax expression in the hippocampus (Figure 3C). More importantly, cleaved caspase-3 level in the hippocampus of db/db mice were significantly increased compared with that in the db/m mice (Figure 3D). These results suggest that diabetes induces excessive neuronal apoptosis by triggering the mitochondrial pathway.

### Diabetes Largely Triggers Elevated ER Stress and Activates the ASK1-JNK1/2 Signaling Pathway in the Hippocampus

**FIGURE 4:**
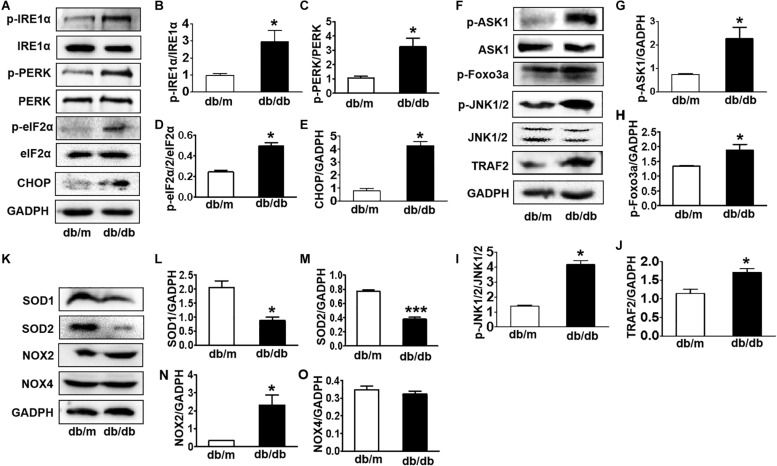
Diabetes largely triggers elevated ER stress and activates the ASK1-JNK1/2 signaling pathway in the hippocampus. **(A–E)** Western blotting and quantitative analysis of p-IRE1α, p-PERK, p-eIF2α, and CHOP expression in the hippocampus of db/m mice and db/db mice. **(F–J)** Western blotting and quantitative analysis of p-ASK1, TRAF2, p-JNK1/2, and p-FoxO3a expression in the hippocampus of db/m mice and db/db mice. **(K–O)** Western blotting and quantitative analysis of SOD1, SOD2, NOX2, and NOX4 expression in the hippocampus of db/m mice and db/db mice. *p < 0.05, ***p < 0.001 vs db/m mice, *n* = 3.

Here, we further detected whether diabetes significantly induces ER stress and triggers the ASK1-JNK1/2 pathway in the hippocampus during DICD. We examined the expression of ER stress markers in the hippocampus and found that the protein levels of p-IRE1α, p-PERK, p-eIF2α, and CHOP were significantly increased in the hippocampus of db/db mice when compared with those in db/m mice (Figures 4A–E). Then, we observed that diabetes remarkably enhances the expression levels of p-ASK1, p-JNK1/2, p-FoxO3a, and TRAF2 in the hippocampus (Figures 4F–J). Furthermore, we had also detected the oxidative stress level (upstream of ASK1-JNK1/2 signaling) and found that diabetes significantly inhibits SOD1 and SOD2 expressions and elevates NOX2 level (Figures 4K–O) in the hippocampus. These studies indicate that diabetes largely triggers elevated ER stress and activates the oxidative stress-ASK1-JNK1/2 signaling pathway in hippocampus.

### Diabetes Enhances the Formation of the IRE1α–TRAF2–ASK1 Complex in the Hippocampus During DICD

**FIGURE 5:**
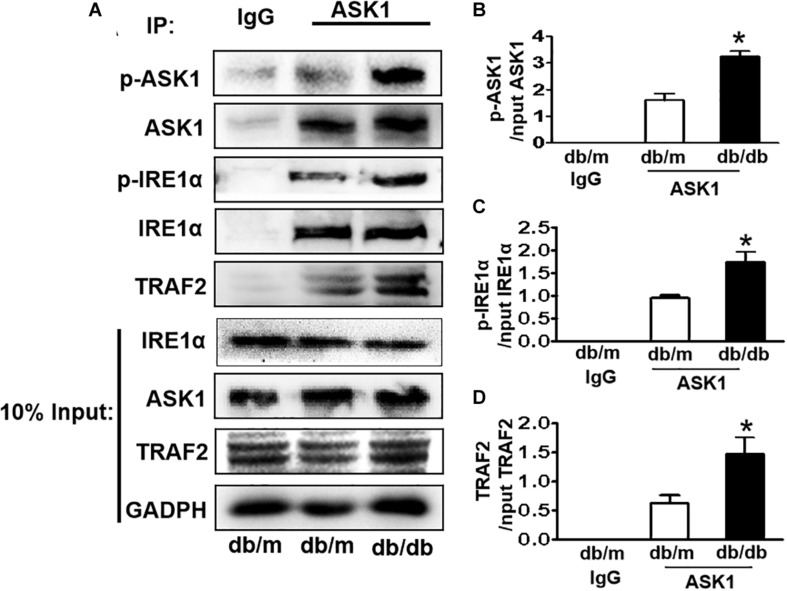
Diabetes enhances the formation of IRE1α–TRAF2–ASK1 complex in the hippocampus during DICD. **(A)** Expression levels of p-IRE1α, TRAF2, and p-ASK1 in ASK1 immunoprecipitates. **(B–D)** The quantitative analysis of p-IRE1α, TRAF2, and p-ASK1 expression was normalized by respective levels in 10% input. Normal rabbit IgG was used as control. *p < 0.05 vs db/m mice, *n* = 3.

IRE1α interacts with TRAF2 and ASK1, forming a proapoptotic signalosome, the IRE1α–TRAF2–ASK1 complex (Urano et al., 2000; Nishitoh et al., 2002), which triggers the mitochondrial apoptotic pathway by activating the proapoptotic Bcl-2 family members Bax and Bak (Hetz et al., 2006; Soustek et al., 2018), suggesting that ER stress and ASK1 signaling have a crosstalk during apoptosis. Here, we performed an IP assay using an ASK1 antibody to test whether diabetes induces IRE1α–TRAF2–ASK1 formation in the hippocampus. The abundance of p-IRE1α, TRAF2, and p-ASK1 in ASK1 immunoprecipitates was significantly increased in the hippocampus of db/db mice compared to those in db/m mice (Figures 5A–D), indicating that diabetes enhances the formation of IRE1α–TRAF2–ASK1 complex in the hippocampus.

### Suppressing ER Stress by 4-PBA Blocks HG-Triggered ASK1-JNK1/2 Signaling Activation and Excessive Apoptosis in SH-SY5Y Cells

**FIGURE 6:**
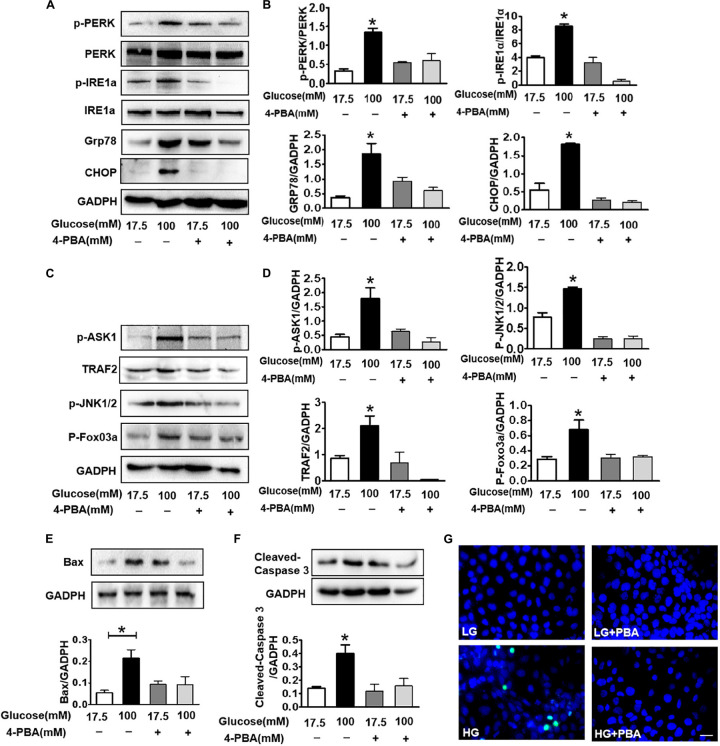
Suppressing ER stress by 4-PBA blocks HG-triggered ASK1-JNK1/2 signaling activation and excessive apoptosis in SH-SY5Y cells. Glucose (100 μM) was used as the HG condition. 4-PBA (2 mM) was used to inhibit ER stress. **(A,B)** Western blotting and quantitative analysis of p-PERK, p-IRE1α, GRP78, and CHOP expression in SH-SY5Y cells under HG with or without 4-PBA. **(C,D)** Western blotting and quantitative analysis of p-ASK1, TRAF2, p-JNK1/2, and p-FoxO3a expression in SH-SY5Y cells under HG with or without 4-PBA. **(E,F)** Western blotting and quantitative analysis of Bax and cleaved caspase-3 expression in SH-SY5Y cells under HG with or without 4-PBA. **(G)** Representative images of the TUNEL assay showing apoptotic cells (green signal) in SH-SY5Y cells under HG with or without 4-PBA. Cell nuclei were stained with DAPI (blue) (scale bar = 15 μm). HG: high glucose; 4-PBA: 4-phenylbutyric acid. *p < 0.05 vs the other group, *n* = 3.

To further confirm that ER stress triggers ASK1-JNK signaling activation and subsequently affects apoptosis, the ER stress inhibitor, 4-PBA, was used to suppress ER stress. Using the CCK8 assay and TUNEL staining, we found that 100 mM glucose treatment for 48 h significantly induced apoptosis in SH-SY5Y cells. Here after, a concentration of 100 mM HG was used as the HG group (Supplementary Figure). Then, it was observed that 4-PBA administration remarkably blocks HG-induced increase in ER stress and inhibits p-PERK, p-IRE1α, GRP78, and CHOP in SH-SY5Y cells (6A,B). Additionally, we assessed the p-ASK1 level and its downstream molecular expression in SH-SY5Y cells under HG with or without 4-PBA. We found that 4-PBA treatment significantly inhibits HG-induced ASK1-JNK1/2 signaling activation as evidenced by the suppression of p-ASK1, TRAF2, p-JNK1/2, and p-FoxO3a expressions (Figures 6C,D). Moreover, 4-PBA treatment remarkably abolished the HG-induced robust expression of Bax, cleaved caspase-3, and robust TUNEL-positive signals in SH-SY5Y cells (Figures 6E–G).

### ASK1 Inhibition Ameliorates the HG-Induced Elevated ER Stress and Excessive Apoptosis in SH-SY5Y Cells

**FIGURE 7:**
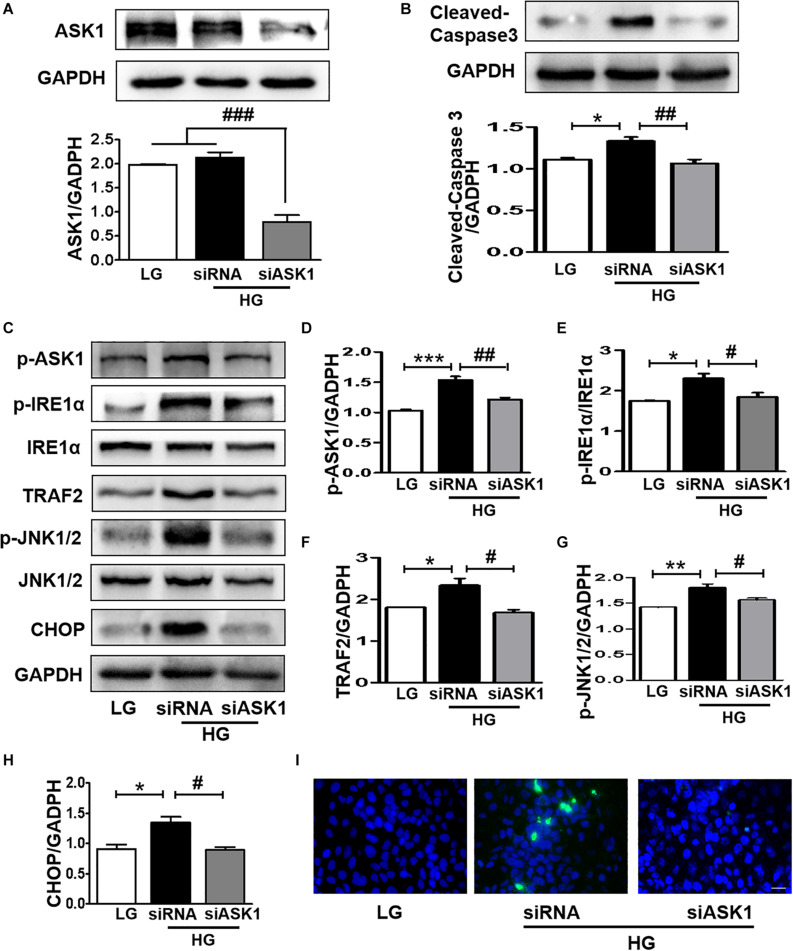
ASK1 inhibition ameliorates HG-induced elevated ER stress and excessive apoptosis in SH-SY5Y cells. ASK1 siRNA was used to inhibit ASK expression and detect the effect of ASK1 signaling on ER stress and apoptosis in SH-SY5Y cells. **(A)** The expression of ASK1 in SH-SY5Y cells after stimulation with ASK1 siRNA. **(B)** Western blotting and quantitative analysis of cleaved-caspase-3 expression in SH-SY5Y cells under HG with or without siASK1. **(C–H)** Western blotting and quantitative analysis of p-ASK1, p-IRE1α, TRAF2, p-JNK1/2, and CHOP expression in SH-SY5Y cells under HG with or without ASK1 siRNA. **(I)** Representative images of the TUNEL assay showing apoptotic cells (green signal) in SH-SY5Y cells under HG with or without ASK1 siRNA. Cell nuclei were stained with DAPI (blue) (scale bar = 15 μm). LG: low glucose; HG: high glucose. *p < 0.05, **p < 0.01, ***p < 0.001 vs the LG group; **^#^***p* < 0.05, **^##^***p* < 0.01, **^###^***p* < 0.001 vs the HG + siASK1 group, *n* = 3.

The current study demonstrated that diabetes significantly induces elevated ER stress, triggers ASK1-JNK1/2 signaling activation, and enhances the formation of the IRE1α–TRAF2–ASK1 complex. Suppression of ER stress remarkably inhibited ASK1-JNK1/2 signaling activation in SH-SY5Y cells under HG conditions. Thus, we had further used siASK1 to inhibit ASK1 expression and verified the reciprocal relationship between ASK1-JNK1/2 pathway and ER stress in SH-SY5Y cells under HG conditions. ASK1 siRNA treatment remarkably reduced ASK1 protein level to 75 and 80% of those in the other two control groups, respectively (Figure 7A). Furthermore, ASK1 inhibition abolished HG-induced increases in cleaved-caspase-3, p-ASK1, TRAF2, p-JNK1/2, p-IRE1α, and CHOP expression (Figures 7B–H). Moreover, robust TUNEL-positive signals of SH-SY5Y cells under HG conditions were ameliorated by ASK1 siRNA treatment (Figure 7I). Taken together, these results indicate that ASK1 inhibition ameliorates HG-induced increases in ER stress and excessive apoptosis in SH-SY5Y cells.

## Discussion

DICD is a serious, chronic diabetes-induced encephalopathy that results in the structural and functional changes in the brain. DICD manifests a series of AD symptoms, including decreased learning and memory ability and impaired language, understanding, and judgment. Due to its serious effect on the lives of diabetic patients, it is very important to explore the precise mechanisms and seek the effective potential therapeutic strategies for DICD. In this study, we confirmed that diabetes remarkably induces cognitive decline of mice with inferior spatial learning and memory function, Aβ_1__–__42_ large deposition, and synaptic dysfunction in the hippocampus. The molecular mechanisms underlying DICD seem to be multifactorial. Accumulating evidence has demonstrated that neurons exert a fundamental role in the modulation of synaptic activities and cognitive processes (Winocur et al., 2005). Hyperglycemia-mediated excessive neuronal apoptosis in the hippocampus is an important causal factor in learning and memory deficits (Ye et al., 2011; Zhao et al., 2012).

The hippocampus is an important functional area of the brain that has three main regions: CA1, CA3, and the dentate gyrus (DG). The hippocampus is essential for the regulation of short-term memory, learning, executive ability, and attention, especially the CA1 region. More importantly, CA1 is also a vulnerable region for external factors, such as hyperglycemia (Ye et al., 2011), and associated cellular stress (Lee et al., 2015) and apoptosis (Zhao et al., 2012) conditions. It has previously been reported that diabetes disturbs the structure and function of neurons, axons, and synapses in the CA1 region (Xiang et al., 2015), and then influences synaptic plasticity and long-term potentiation (LTP) formation (Bliss and Collingridge, 1993). In the current study, excessive neuronal apoptosis and synaptic dysfunction were observed in the CA1 region of hippocampus from db/db mice, which is consistent with prior study.

Hyperglycemia with resulting elevated cellular stress is the major mediator of diabetes-associated onset of complications (Wu et al., 2015, 2018). Multiple studies have shown that the induction of ER stress is responsible for the pathogenetic progression of diabetes-associated neuropathy (Lupachyk et al., 2013; Zhang et al., 2013; Wu et al., 2015). In this study, we found that diabetes significantly led to elevated ER stress by triggering IRE1α activity, which is the main mediator of ER-associated apoptosis. Activated IRE1α during ER stress may lead to IRE1α autophosphorylation and activation of its RNase activity, consequently triggering apoptosis. Although the accumulation of unfolded/misfolded proteins in the ER triggers initial or transient IRE1α activation, prolonged IRE1α activation requires an additional mechanism to result in apoptosis (Walter and Ron, 2011), suggesting that ASK1 is one of these mechanisms that induces prolonged IRE1α activation. Our study and other studies have verified that ASK1 is a critical component for inducing IRE1α activity that leads to ER stress (Nishitoh et al., 2002; Wang et al., 2015a).

It has been reported that the IRE1α signalosome increases Txnip, a new proapoptotic factor, and contributes to ASK1 activation (Wang et al., 2015a). Our findings also showed that diabetes induces ASK1-JNK1/2 signaling in the hippocampus and leads to apoptosis through the mitochondrial pathway. Moreover, diabetes significantly enhances the formation of the IRE1α–TRAF2–ASK1 complex in the hippocampus. These studies indicate that independent of ER stress, IRE1α exerts its proapoptotic effect by activating the ASK1-JNK1/2 pathway, supporting the hypothesis that IRE1α activity and ASK1 pathway activation has a reciprocal causal relationship during DICD. Additionally, diabetes-induced oxidative stress in the hippocampus of rats has been previously reported as a factor that contributes to cognitive dysfunction (Wang et al., 2010; Tabatabaei et al., 2017; Farbood et al., 2019) with evidence of synaptic damage (Fukui et al., 2001, 2002) and excessive neuronal apoptosis (Farbood et al., 2019; Nazarnezhad et al., 2019; Yan et al., 2019). The ASK1-JNK1/2 pathway, the important downstream of oxidative stress, may be the major mechanism for oxidative stress-mediated apoptosis during DICD, suggesting that cellular stress does not independently affect DICD development.

Oxidative stress and ER stress are the two main caused events during HG-induced neuronal apoptosis. Previous studies have established the causal link between HG-induced oxidative stress and ER stress (Chen et al., 2018). It has been reported that HG increases the production of ROS and impairs endogenous antioxidant enzymes, leading to oxidative stress. Increased glucose flux disrupts mitochondrial function by impairing the mitochondrion integrity and, thus, increases mitochondrial ROS (Zhong et al., 2016). Oxidative stress can induce ER stress. The previous study showed that the SOD mimetic Tempol blocked HG-induced ER stress and UPR (Chen et al., 2018). In addition, H_2_O_2_ triggered ER stress and UPR (Chen et al., 2018). These evidences further reinforce the causal role of oxidative stress in ER stress and UPR induction under HG conditions. The oxidative stress–ER stress–UPR–apoptosis pathway mediates the role of HG on neuron.

Although these studies have clearly verified the role of ASK1-JNK1/2 signaling and its reciprocal causation with ER stress in the induction of apoptosis during DICD, there are some shortcomings to our current experimental strategies. Here, ASK1 siRNA was used to verify the effect of ASK1 on IRE1α, rather than *ASK1* gene knockout mice and directly reflects the effect of the *ASK1* gene on the cognitive behavior of diabetic mice. Thus, conditional deletion of the *ASK1* gene is needed to reveal the role of ASK1 in DICD development in future studies. IRE1α exerts dual functions of proapoptotic kinase and RNase activity. IRE1α kinase directly triggers its RNase activity (Calfon et al., 2002). Recently, it has been reported that the RNase activity of IRE1α influences the expression of proapoptotic factors and is involved in the proapoptotic process by cleaving miRs (Upton et al., 2012). In this study, we did not further explore the relationship between RNase activity and the proapoptotic kinase activity of IRE1α during neuronal apoptosis, or the role of ASK1 on this process. This is the other main shortcoming that needs to be further explored.

## Conclusion

**FIGURE 8:**
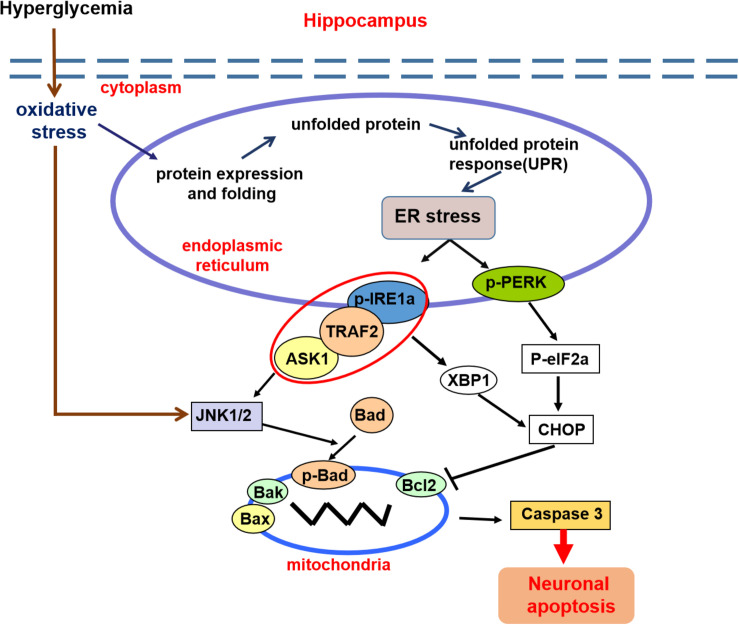
A schematic diagram showing the crosstalk of the ASK1-JNK1/2 pathway and ER stress during DICD. Diabetes significantly induces ER stress and triggers ASK1-JNK1/2 pathway activation in the hippocampus, which leads to excessive neuronal apoptosis and contributes to DICD development. More importantly, diabetes enhances the formation of the IRE1α–TRAF2– ASK1 complex, which promotes the crosstalk of ER stress and the ASK1-JNK1/2 signaling pathway during DICD.

In summary, these findings demonstrated that diabetes significantly induces cognitive dysfunction of mice. The current mechanistic study found that diabetes remarkably activates the ASK1-JNK1/2 pathway in the hippocampus, which is mutually regulated with ER stress via the formation of the IRE1α–TRAF2–ASK1 complex during DICD (Figure 8). Our current findings support the idea that activation of the ASK1-JNK1/2 pathway and elevated ER stress are the interdependent and reciprocal causation during DICD, suggesting that ASK1 is a potentially important target for the treatment of DICD.

## Data Availability Statement

All datasets generated for this study are included in the article/Supplementary Material.

## Ethics Statement

The animal study was reviewed and approved by Laboratory Animal Ethics Committee of Wenzhou Medical University.

## Author Contributions

YW and YY researched data and wrote the manuscript. CW participated in data analyses and in the writing of the manuscript. TJ, BW, JXio, YiL, PZ, YaL, JXu, and XL researched data. JXia conceived the project, designed the experiments, and wrote the manuscript. All authors have approved the final version of the manuscript.

## Conflict of Interest

The authors declare that the research was conducted in the absence of any commercial or financial relationships that could be construed as a potential conflict of interest.

## Abbreviations

ASK1: apoptosis signal-regulating kinase 1
BSA: bovine serum albumin
CA1: cornu ammon1
DE: diabetic encephalopathy
DG: dentate gyrus
DICD: diabetes-induced cognitive decline
ER: endoplasmic reticulum
HG: high glucose
IRE1 α: inositol-requiring enzyme 1 α
LTP: long-term potentiation
PBS: phosphate-buffered saline
PERK: protein kinase RNA-like ER kinase
PFA: paraformaldehyde
TRAF2: TNF receptor-associated factor 2
UPR: unfolded protein response.
