# Corrigendum: The Reciprocal Causation of the ASK1-JNK1/2 Pathway and Endoplasmic Reticulum Stress in Diabetes-Induced Cognitive Decline

**DOI:** 10.3389/fcell.2021.639486

**Published:** 2021-02-24

**Authors:** Yanqing Wu, Yuan Yuan, Chengbiao Wu, Ting Jiang, Beini Wang, Jun Xiong, Peipei Zheng, Yiyang Li, Jingyu Xu, Ke Xu, Yaqian Liu, Xiaokun Li, Jian Xiao

**Affiliations:** ^1^The Institute of Life Sciences, Engineering Laboratory of Zhejiang Province for Pharmaceutical Development of Growth Factors, Biomedical Collaborative Innovation Center of Wenzhou, Wenzhou University, Wenzhou, China; ^2^Research Units of Clinical Translation of Cell Growth Factors and Diseases Research of Chinese Academy of Medical Science, School of Pharmaceutical Science, Wenzhou Medical University, Wenzhou, China; ^3^Clinical Research Center, Affiate Xiangshang Hospital, Wenzhou Medical University, Wenzhou, China

**Keywords:** diabetes-induced cognitive decline (DICD), hippocampus, neuronal apoptosis, apoptosis signal-regulating kinase 1 (ASK1), endoplasmic reticulum (ER) stress

In the original article, there were some mistakes in “Figure 1G, (Figures 2A, C, and D) and Figure 6B” as published. The image of representative swimming track of db/m mice in the probe trial (24 h after training) (Figure 1G), and the images of immunohistochemical staining of Aβ_1−42_ and immunofluorescence staining of p-Tau in the CA1 region of db/m mice (Figure 2A) should be replaced with appropriate images. The last result of quantitative analysis in Figure 6B should be the quantitative analysis of CHOP. The labels in Figures 2C and D of Synapthysin-1 and Synapsin should be Synapthysin and Synapsin-1. The corrected Figures appears below.

**Figure 1:**
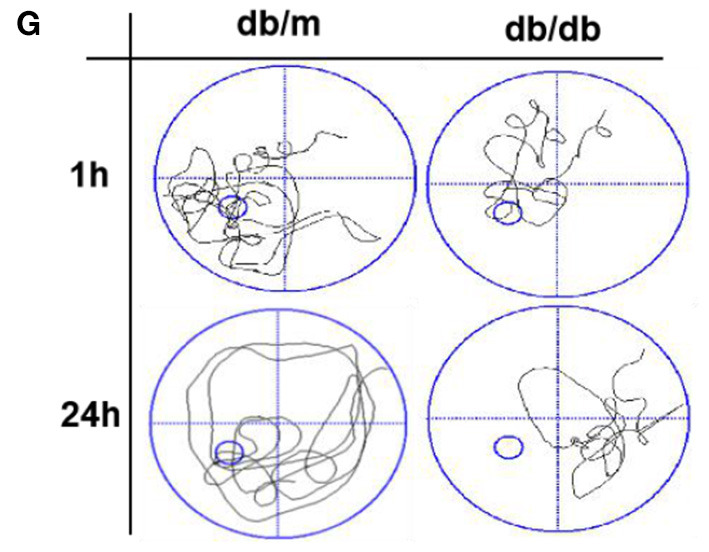
Diabetes significantly induces cognitive decline of mice. **(A)** The learning curve of the training period of mice during six blocks in the Morris water maze test. **(B)** Representative swimming track of mice at block 1 and block 6 during the training period. **(C)** Number of crossings over the original platform location of mice in the probe trial (1 h after training). **(D)** Latency to find the platform of mice in the probe trial (1 h after training). **(E)** Number of crossings over the original platform location of mice in the probe trial (24 h after training). **(F)** Latency to find the platform of mice in the probe trial (24 h after training). **(G)** Representative swimming track of mice in the probe trial (1 and 24 h after training). **(H)** Percentage of residence time in each quadrant. The quadrant with the platform was designated as TQ and the quadrant from which the mice started their swimming was designated as OP for “opposite”. The quadrant on the left side of OP was designated as AL for “adjacent left” and the quadrant on the right side of OP was designated as AR for “adjacent right”. *p < 0.05, **p < 0.01 vs db/m, *n* = 10.

**Figure 2:**
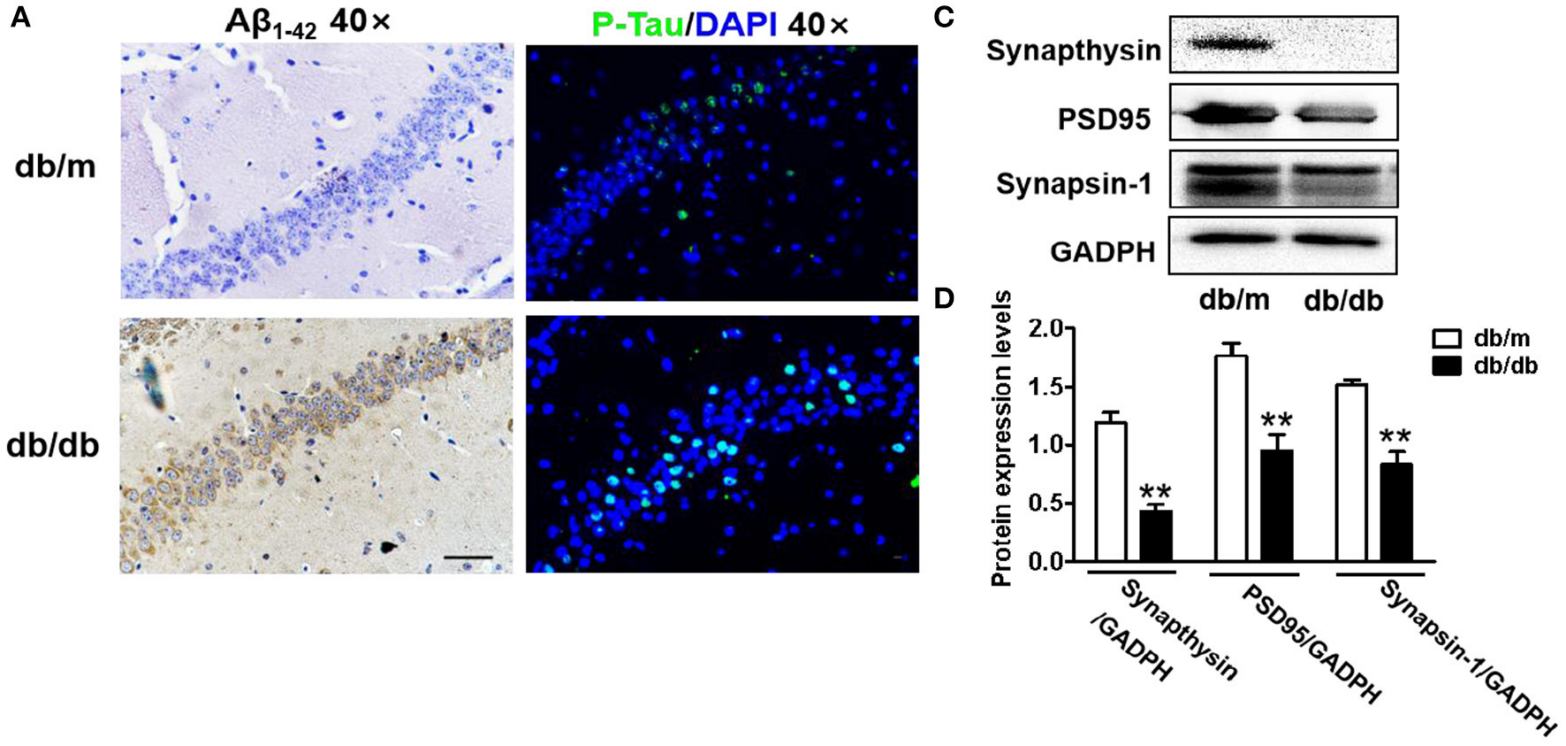
Diabetes significantly leads to morphological changes, Aβ_1-42_ deposition, hyperphosphorylation of Tau and synaptic dysfunction in the hippocampus during DICD. **(A)** The representative images of H&E staining, Nissl staining, immunohistochemical staining of Aβ_1-42_, and immunofluorescence staining of p-Tau in the CA1 region of the hippocampus of db/m mice and db/db mice (scale bar = 15 μm). **(B)** Westere blotting and quantitative analysis of Aβ_1-42_ in the hippocampus of db/m mice and db/db mice. **(C,D)** Western blotting and quantitative analysis of synaptic function-related protein expression (PSD95, synaptophysin, and synapsin-1) in the hippocampus of db/m mice and db/db mice. *p < 0.05, **p < 0.01 vs db/m mice, *n* = 3.

**Figure 6:**
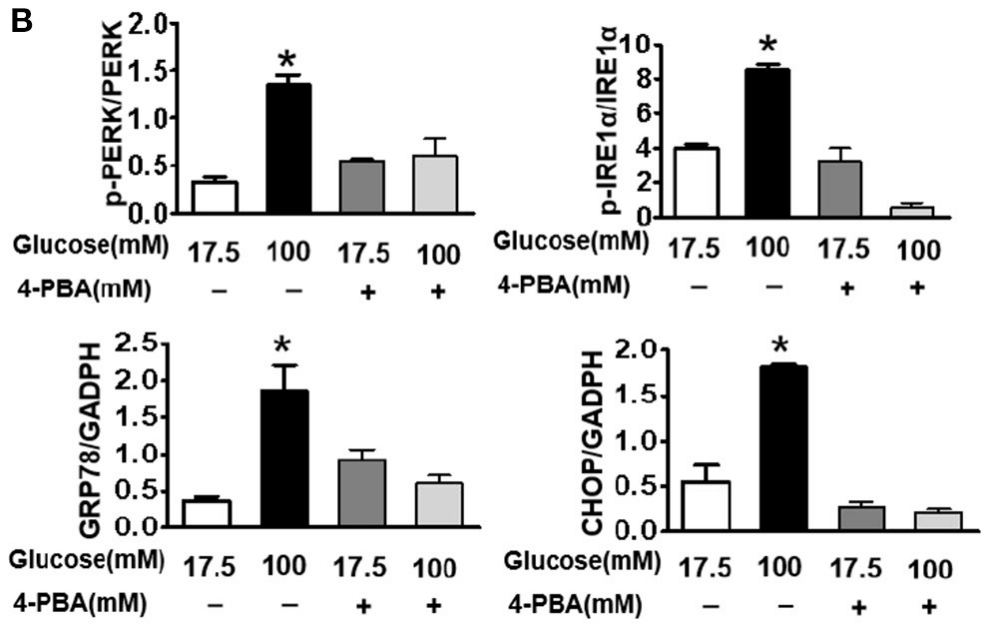
Suppressing ER stress by 4-PBA blocks HG-triggered ASK1-JNK1/2 signaling activation and excessive apoptosis in SH-SY5Y cells. Glucose (100 μM) was used as the HG condition. 4-PBA (2 mM) was used to inhibit ER stress. **(A,B)** Western blotting and quantitative analysis of p-PERK, p-IRE1α, GRP78, and CHOP expression in SH-SY5Y cells under HG with or without 4-PBA. **(C,D)** Western blotting and quantitative analysis of p-ASK1, TRAF2, p-JNK1/2, and p-FoxO3a expression in SH-SY5Y cells under HG with or without 4-PBA. **(E,F)** Western blotting and quantitative analysis of Bax and cleaved caspase-3 expression in SH-SY5Y cells under HG with or without 4-PBA. **(G)** Representative images of the TUNEL assay showing apoptotic cells (green signal) in SH-SY5Y cells under HG with or without 4-PBA. Cell nuclei were stained with DAPI (blue) (scale bar = 15 μm). HG: high glucose; 4-PBA: 4-phenylbutyric acid. *p < 0.05 vs the other group, *n* = 3.

The authors apologize for this error and state that this does not change the scientific conclusions of the article in any way. The original article has been updated.

