# Supplementary material for: The Reciprocal Causation of the ASK1-JNK1/2 Pathway and Endoplasmic Reticulum Stress in Diabetes-Induced Cognitive Decline
Source: Front Cell Dev Biol. 2020 Jul 17;8:602. doi: 10.3389/fcell.2020.00602 (PMC7379134; doi:10.3389/fcell.2020.00602)
Supplement: Supplementary file 1 [file Image_1.pdf]

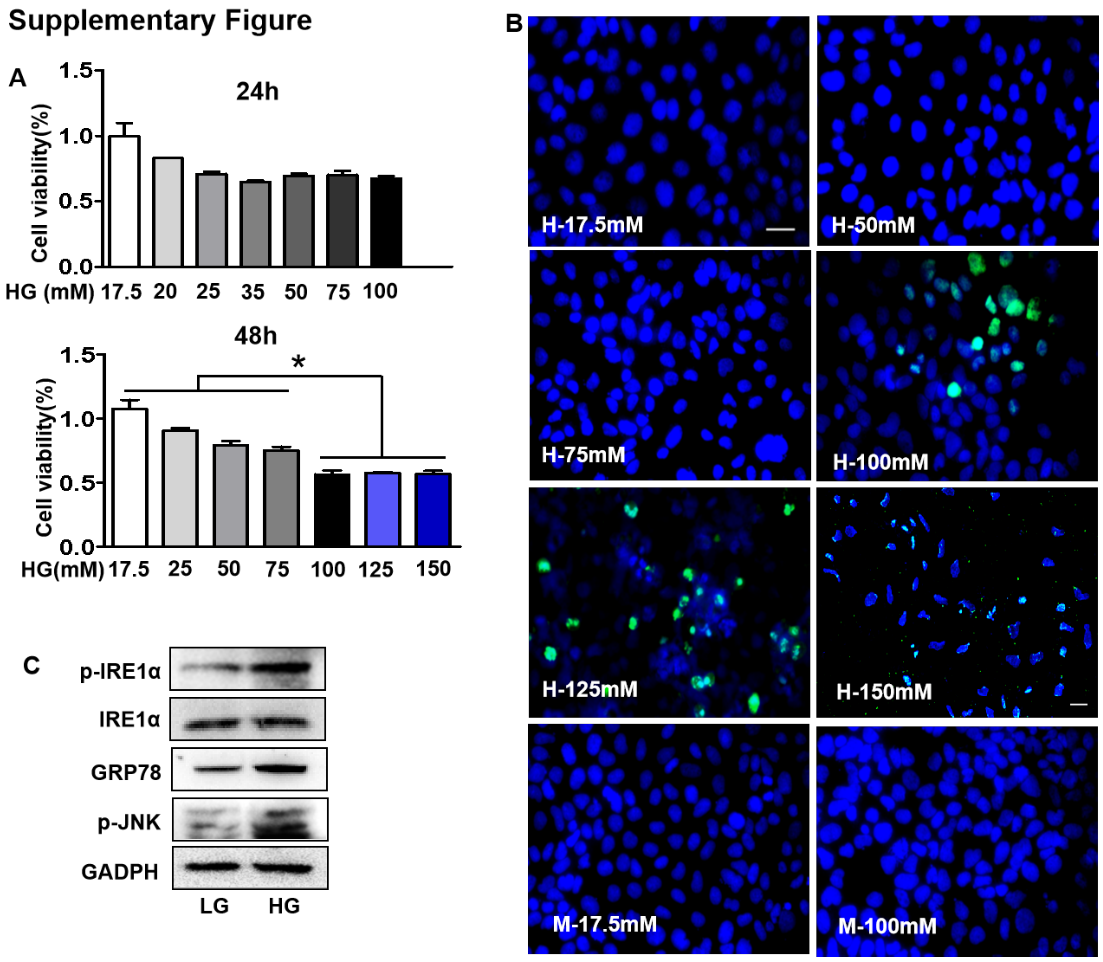

Supplementary Figure. The effect of different concentrations of HG on apoptosis in SH-SY5Y cells. (A) Cell viability of SH-SY5Y cells treating with different concentrations of HG for 24h or 48h; (B) TUNEL staining of SH-SY5Y cells treating with different concentration of HG or mannitol for 48h; (C) Western blotting p-IRE1α, GRP78, and p-JNK1/2 expressions in SH-SY5Y cells treating with 100mM HG for 48h. HG: high glucose, M: mannitol.
